# The Place of Targeted Agents in the Treatment of Elderly Patients with Metastatic Colorectal Cancer

**DOI:** 10.3390/cancers7010439

**Published:** 2015-03-13

**Authors:** Alexios Matikas, Natalia Asimakopoulou, Vassilis Georgoulias, John Souglakos

**Affiliations:** Department of Medical Oncology, University General Hospital of Heraklion, Voutes, PO Box 1352, 71110 Heraklion, Greece; E-Mails: almatikas@gmail.com (A.M.); nataliasimak@gmail.com (N.A.); georgsec@med.uoc.gr (V.G.)

**Keywords:** colorectal cancer, elderly, bevacizumab, cetuximab, panitumumab, targeted

## Abstract

Despite the high prevalence of colorectal cancer in a continuously aging population and the substantial advances in the treatment of metastatic disease during the past decade, the treatment of elderly patients with advanced, unresectable or metastatic colorectal cancer is a clearly unmet need. Since older patients are under-represented or even excluded from randomized trials, the evidence that oncologists use as guidance is weak. However, small prospective studies, pooled analyses and observational studies show that combination approaches are safe, efficacious and feasible in the geriatric population with metastatic colorectal cancer. The use of biologic agents targeting angiogenesis and the epidermal growth factor receptor, which have been shown to clearly improve outcomes in multiple prospective trials in patients with advanced colorectal cancer, is a vital component of the aforementioned combination approaches. Herein, we review all available data concerning the management of elderly patients with these agents and underscore the differences between this age subgroup and younger patients.

## 1. Introduction

Colorectal cancer (CRC) is a disease of the elderly. Approximately 60% of cases are diagnosed in patients over 65 years old, with a median age of 69 at diagnosis [1]. Combined with the growing elderly population, the emerging CRC epidemic in geriatric populations is a major health concern, which needs to be addressed. However, there are numerous challenges that need to be overcome. First of all, diminishing organ function and reserve with age, comorbidities and polypharmacy, lack of social support and concerns about adherence have an impact on decision making. Indeed, data show that elderly patients with metastatic disease (mCRC) are less likely to receive chemotherapy [2]. Furthermore, evidence that could guide the treatment of elderly patients mCRC are sparse; this patient group is often under-represented in prospective trials or their representation is skewed, as patients of poorer functional status are excluded and only recently have trials of elderly patients only been published in the literature. Oncologists need to take into account these factors when tailoring the management of the older patient as the goals of palliation, prolongation of life and improvement of the quality of life are confounded by the simultaneous presence of co-morbidities and limited life expectancy.

In an effort to quantify the aforementioned concerns regarding the safety of cytotoxic chemotherapy in this population group and to stratify patients not according to their calendar age, but their biologic age, numerous tools have been developed. One of the most widely used is the Comprehensive Geriatric Assessment (CGA), which measures medical co-morbidities, psychosocial status, geriatric syndromes, functional status regarding activities of daily living (ADL) and instrumental activities of daily living (IADL), mood disorders and cognition [3]. Based on CGA, models that predict excess toxicity from chemotherapy in elderly patients have been developed [4], which, if validated in prospective trials, will be of significant value in the decision making process.

The significant progress in the management of mCRC with the introduction of multiple effective cytotoxics, such as oxaliplatin and irinotecan, and targeted agents, such as bevacizumab, cetuximab, panitumumab, aflibercept and regorafenib, has greatly improved outcomes; the reported overall survival (OS) in recent trials often exceeds 30 months. Whether these advances can also be applied to geriatric patients is a matter of debate. The use of combination chemotherapy in elderly patients has been shown to be effective in randomized trials and pooled analyses [5,6,7]. However, the use of biologic agents specifically in this subgroup has not been formally addressed in randomized, placebo-controlled trials, with a single exception. Herein, we review the available published data concerning the use of agents that target angiogenesis and the epidermal growth factor receptor (EGFR) in elderly patients with mCRC.

## 2. Agents that Target Angiogenesis

### 2.1. Bevacizumab

Bevacizumab is a recombinant humanized IgG antibody that binds vascular endothelial growth factor-A (VEGF-A), thus inhibiting angiogenesis. Multiple trials have shown that adding bevacizumab to a chemotherapy backbone improves outcomes in patients with mCRC [8,9,10], and it also extends survival when continued after the patient has progressed on bevacizumab [11]. Even though the use of bevacizumab is supported in current practice guidelines regarding the management of mCRC [12], there is a paucity of data supporting its use in elderly patients, mainly due to concerns of increased toxicity in this vulnerable age group.

Initial evidence concerning the role of bevacizumab when treating this patient subgroup came from observational studies. In the BRiTE observational cohort study, 896 patients over 65 years old received a combination of chemotherapy with bevacizumab. Progression-free survival (PFS) was similar across age groups (9.8 months < 65, 9.6 months 65–75, 10 months 75–80 and 8.6 months > 80 years old), but OS declined with age, possibly reflecting death from other causes. Treatment-related adverse events were similar with the exception of arterial thrombosis, which was noted for approximately 4% of patients over 75 years old and 1.4% under that cut-off. However, rates of arterial thrombotic events (ATE) were influenced more by performance status (PS), the presence of arterial hypertension or of a history of ATE and the use of anticoagulation, rather than age [13]. Similar results between younger and older patients with mCRC were also reported by the Bevacizumab Expanded Access Trial (BEAT), where 33% of the 1965 enrolled patients were over 65 years old and 7% were over 75 [14]. A third observational study reported data from 2526 patients with mCRC over 65 years old from the Surveillance, Epidemiology and End Results (SEER) database; all of the patients received a chemotherapy doublet, and 36% also received bevacizumab. The addition of bevacizumab improved OS, but this effect was mainly observed in patients treated with the now outdated IFL regimen [15]. The fourth observational study was from the Czech registry and compared outcomes between 2126 mCRC patients < 65 years old *versus* 1061 > 65 treated with bevacizumab-based combinations; PFS and OS were similar, as were adverse event rates, with the exception of hypertension [16]. It should be noted however that observational studies, while they may reflect every day practice, suffer from inherent hazards: selection bias, as it is possible that only patients deemed fit enough received bevacizumab; and the treatment-related toxicities may be underreported, as is the case in prospective clinical trials.

The administration of bevacizumab to elderly patients with mCRC has also been evaluated in subgroup analyses and pooled analyses of prospective randomized trials. The AGITG MAX trial was a three-arm trial that compared capecitabine, capecitabine plus bevacizumab and capecitabine, bevacizumab and mitomycin C. Among the 99 patients over 75 years old (88% of whom had a PS of 0–1), bevacizumab improved PFS with no added toxicity [17]. Moreover, two pooled analyses of previous randomized trials of bevacizumab reported results in older populations. The first one analyzed data from two trials, including 439 patients, whereas the second one had data from four trials (including the two from the first pooled analysis), including 1142 patients. The conclusion in both analyses was that adding bevacizumab improved overall and progression-free survival, and adverse event rates were similar to the ones reported in the original trials [10,18].

The most robust data concerning the efficacy and safety of bevacizumab in elderly patients are derived from prospective trials. AVEX is the only prospective, randomized, phase 3 trial that addresses the role of bevacizumab in an elderly-only population with mCRC. Two hundred eighty patients aged 70–87 (median 76), with a PS of 0–2, who were deemed ineligible for combination chemotherapy by their treating physician, were randomized to receive capecitabine monotherapy *versus* capecitabine and bevacizumab combination. PFS (9.1 *versus* 5.1 months), response rates (19% *versus* 11%) and disease control rate (74% *versus* 58%) all favored the combination, and the differences were statistically significant. However, the difference in OS did not reach statistical significance (20.7 *versus* 16.7 months). Moreover, treatment-related serious adverse events were more common in the combination group (14% *versus* 8%), and dose modifications were required more frequently (41% *versus* 26%) [19].

The combination of bevacizumab with an oxaliplatin-based doublet was examined in two phase 2 trials. In the first one conducted by the Hellenic Oncology Research Group, 46 patients aged > 70 years old with treatment naive mCRC received a modified version of XELOX/bevacizumab. The reported response rate was 46.8%; PFS was 7.9 months, and OS 20.1 months; these results compare favorably to those reported in younger patients. The integration of the Comprehensive Geriatric Assessment in the trial, which enrolled fit and vulnerable patients, resulted in very low toxicity rates and high compliance; grade 3–4 toxicities included asthenia (4.2%), diarrhea (6.3%) and neurotoxicity (2.1%) [20]. The other published trial conducted by the Spanish GEMCAD group (BECOX trial) evaluated the XELOX-bevacizumab combination in 68 patients over 70 years old. Response rates were similar to the previous trial (46%), and the reported time to progression was 11.1 months. Common (over 10%) grade 3–4 adverse events were diarrhea and asthenia; bevacizumab-related toxicity included deep venous thrombosis and pulmonary embolism, in 6% and 4% of the patients, respectively [21].

The above results underscore the efficacy and safety of bevacizumab combinations in appropriately selected elderly patients with mCRC. The feasibility of an approach based on risk stratification facilitated by powerful tools, such as the CGA, is clearly shown in these trials. The next logical steps would be to evaluate triplet combinations in placebo-controlled trials conducted in fit and vulnerable elderly patients and to address the question of whether a fluoropyrimidine with or without bevacizumab improves outcome in frail patients, as assessed by CGA.

### 2.2. Aflibercept

Aflibercept is a recombinant fusion protein that acts as a high affinity ligand trap for VEGF-A, VEGF-B and placental growth factor (PlGF), thus preventing its targets from binding to their receptors. In the phase 3 VELOUR trial, adding aflibercept to FOLFIRI after progression on an oxaliplatin-based first line regimen significantly improved response rates, PFS and OS compared to placebo, regardless of whether the patients had previously received bevacizumab. The median age of the patient population was 61 years (range: 19–86) [22]. In a separately reported subgroup analysis, efficacy and toxicity were similar across age groups [23]. Although the results imply that aflibercept is a valuable option for elderly patients in the second line setting, it is not known whether this approach improves results compared to continuation of bevacizumab beyond progression.

### 2.3. Regorafenib

Regorafenib is a novel oral tyrosine kinase (TK) inhibitor, which inhibits several TKs involved in angiogenesis (VEGFR 1–3), oncogenesis (KIT, RET, RAF, BRAF) and the tumor microenvironment (PDGFR, FGFR). It is approved for use in patients refractory to all other available agents based on the results of the phase 3 CORRECT trial, where, compared to placebo, regorafenib significantly improved PFS (1.9 *versus* 1.7 months) and OS (6.4 *versus* 5.0 months) [24]. However, in patients > 65 years old, regorafenib did not improve OS (HR 0.86, 95% CI 0.61–1.19). Differences in adverse events between different age groups were not reported. Since patients enrolled in the CORRECT trial were up to 69 years old and taking into account the OS results, the use of regorafenib in elderly patients with refractory mCRC cannot be recommended as the standard of care.

## 3. Agents that Target the Epidermal Growth Factor Receptor

EGFR activation is a key point for a variety of processes involved in cancer cell growth, proliferation, angiogenesis and invasion [25]. Inhibition of EGFR-mediated signaling with the use of monoclonal antibodies that target the extracellular domain of the receptor has been shown to improve outcomes in patients with mCRC lacking mutations in the RAS genes (RAS wild-type, WT) [26,27,28].

### Cetuximab

Cetuximab is a chimeric monoclonal antibody that competitively binds to the extracellular domain of EGFR with a higher affinity than its endogenous ligands, blocking EGFR-driven signaling, resulting in inhibition of cell growth and induction of apoptosis. Available data concerning its efficacy and safety in elderly patients with mCRC are derived from retrospective studies, pooled analyses and small prospective trials. In a retrospective study of 56 patients aged over 70 years old with heavily pretreated KRAS WT mCRC who were treated with cetuximab with or without irinotecan, the median PFS was 4.4 months and the median OS 16.0 months. Diarrhea (20% grade 3–4) and skin rash (11% grade 3) were the most frequent adverse events [29]. Furthermore, in a German observational study of 657 patients with mCRC who received cetuximab, 305 of the patients were over 65 years old. Efficacy outcomes and the frequency of adverse events were found to be similar across the two age groups [30].

The administration of cetuximab in elderly patients with mCRC has also been evaluated in subgroup analyses and pooled analyses of large prospective trials. In a Canadian phase 3 trial, 572 pretreated patients with mCRC positive for EGFR expression via immunohistochemistry were randomized to receive either cetuximab or best supportive care alone. Forty one percent of the patients were over 65 years old. OS was similar between the age subgroups, as was the magnitude of benefit derived from cetuximab [31]. Finally, in a pooled analysis of the OPUS [27] and CRYSTAL [26] trials presented in abstract form, the administration of cetuximab to patients over 70 years old was shown to improve OS (23.3 *versus* 15.1 months), and although adverse events were more frequent in the cetuximab cohort, they were not different compared to the younger cohort [32].

In addition, three prospective phase 2 trials concerning the use of cetuximab in older patients have been published. In a trial conducted by the Spanish TTD group, cetuximab monotherapy was compared to best supportive care in 41 previously untreated, molecularly unselected patients aged 70 or older. ORR was 14.6%, PFS 2.9 months and OS 11.4 months [33]. In another trial by the same group, cetuximab was combined with capecitabine in previously untreated patients. Sixty six patients were enrolled; 29 were KRAS WT. The response rate in this subgroup was 48.3% and the PFS 8.4 months [34]. Finally, in the previously-treated setting, cetuximab in combination with irinotecan was found to be safe in 49 patients over 65 years old and resulted in a median PFS of four months and OS of seven months [35].

The above results clearly illustrate the efficacy and safety of cetuximab monotherapy and combinations in elderly patients with mCRC, since age does not seem to influence outcomes. However, it is currently not known whether cetuximab in combination with a chemotherapy doublet, which is standard treatment in younger patients, is well tolerated in this age subgroup. Furthermore, phase 3 elderly-specific trials of cetuximab have yet to be completed, meaning that robust data are lacking.

Panitumumab: Panitumumab is a fully humanized monoclonal antibody directed against the EGFR. There are no prospective trials that specifically address the use of panitumumab in older patients with mCRC. In a phase 3 trial of panitumumab *versus* best supportive care alone in heavily pretreated patients, panitumumab resulted in similar improvements in PFS regardless of whether the patients were over or under 65 years old [36].

## 4. Novel Approaches

Hailed as the breakthrough of the year 2013 by Science [37], cancer immunotherapy has taken the field of medical oncology by storm. Immune checkpoint blockade with monoclonal antibodies targeted against cytotoxic T-lymphocyte antigen 4 (CTLA-4, otherwise known as CD152), like ipilimumab, and programmed death protein 1 (PD-1) and its ligand PD-L1, like nivolumab and pembrolizumab, have been shown to effectively induce host immune response against the tumor, and several have already been approved for use in metastatic melanoma and are in advanced clinical trials in non-small cell lung cancer and other tumors [38,39,40]. The combination of nivolumab with ipilimumab is being compared to nivolumab monotherapy in the Checkmate 142 phase 2 trial in patients with mCRC (NCT02060188), and pembrolizumab is being tested in metastatic tumors with microsatellite instability, including colorectal cancer (NCT01876511). Another potential form of targeted therapy in mCRC is cancer vaccines. Although none have been approved for use, several are being pursued in clinical trials. The most advanced is Imprime PGG, which exerts its antitumor effect via neutrophil priming and is combined with cetuximab in a phase 3 trial (NCT01309126). Several others are in earlier clinical trials, including an antibody that targets the carcinoembryonic antigen tested in the adjuvant setting of stage III CRC. Finally, another novel targeted treatment for patients with mCRC is the use of oncolytic viruses, such as Reolysin, which targets cancer cells with an activated RAS pathway and is being tested in a phase 2 trial combined with standard treatment (FOLFOX and bevacizumab, NCT01622543). Whether any of these novel treatments prove to improve outcomes in randomized trials in patients with mCRC remains to be seen.

## 5. Discussion

Available data regarding the management of older patients with mCRC indicate that they derive clinical benefit from currently approved agents and combinations. The results of published trials are briefly summarized in Table 1. However, careful interpretation is warranted, as published non-randomized studies are plagued with bias due to the subjective nature of the selection of patients deemed fit enough to receive chemotherapy. Furthermore, the under-representation of elderly patients in randomized clinical trials warrants careful interpretation of their results and conclusions, as they may not always be applicable to this frail population, which frequently has severe co-morbidities. These pitfalls are described in detail in recent reviews regarding the treatment of the older patient with mCRC and are recognized in the consensus guidelines of the International Society of Geriatric Oncology [41,42,43].

The optimization of the treatment strategies will be based on three pylons: the design and conduct of elderly-specific prospective randomized trials; the integration of CGA as an objective tool capable to guide risk stratification and decision making and the establishment of more appropriate study endpoints for geriatric patients, such as time to symptom deterioration or the time that quality of life is maintained above predetermined thresholds. These changes will enable oncologists to personalize the management of older patients with mCRC and improve outcomes, while reducing treatment-related toxicities.

**Table 1:** Summary of prospective trials evaluating biologic agents specifically in older patients with mCRC.

Trial	Phase	N	Regimen	Results
Cunningham *et al*. [19]	3	280	Cap *vs.* Cap/Bev	PFS: 5.1 *vs.* 9.1, RR 10% *vs.* 19%, OS 16.7 *vs.* 20.7 (NS)
Vamvakas *et al.* [20]	2	46	XELOX/Bev	RR: 46%, PFS: 7.9, OS: 20.1
Feliu *et al.* [21]	2	68	XELOX/Bev	RR: 46%, TTP: 11.1
Sastre *et al.* [33]	2	41	CTX	RR: 14.6%, PFS 2.9, OS 11.1
Sastre *et al.* [34]	2	66	Cap + CTX	RR: 48.3%, PFS: 8.4 (in KRAS WT)
Abdelwahab *et al.* [35]	2	49	CPT + CTX	PFS 4, OS 7

Cap: capecitabine; Bev: bevacizumab; PFS: progression-free survival; RR: response rate; OS: overall survival; NS: not significant; XELOX: capecitabine + oxaliplatin; TTP: time to progression; CTX: cetuximab; CPT: irinotecan; survival is reported in months.

## 6. Conclusions

In conclusion, we present all of the relevant published data regarding the use of biologic agents in the treatment of elderly patients with mCRC, and we briefly review novel approaches that may impact treatment strategies in the near future. The conduct of elderly-specific randomized trials remains of paramount importance, and treating physicians are encouraged to enroll patients, whenever possible, in well-designed prospective trials that will help answer the clearly unmet need of the optimal medical management of this frail patient subgroup.
